# Impact of skeletal muscle volume on patients with BCLC stage‐B hepatocellular carcinoma undergoing sorafenib therapy

**DOI:** 10.1002/cam4.5810

**Published:** 2023-03-23

**Authors:** Issei Saeki, Takahiro Yamasaki, Yurika Yamauchi, Tomokazu Kawaoka, Shinsuke Uchikawa, Akira Hiramatsu, Hiroshi Aikata, Kazufumi Kobayashi, Takayuki Kondo, Sadahisa Ogasawara, Tetsuhiro Chiba, Reo Kawano, Kazuaki Chayama, Naoya Kato, Taro Takami

**Affiliations:** ^1^ Department of Gastroenterology and Hepatology Yamaguchi University Graduate School of Medicine Yamaguchi Japan; ^2^ Department of Oncology and Laboratory Yamaguchi University Graduate School of Medicine Yamaguchi Japan; ^3^ Department of Gastroenterology and Metabolism, Graduate School of Biomedical and Health Sciences Hiroshima University Hiroshima Japan; ^4^ Department of Gastroenterology, Graduate School of Medicine Chiba University Chiba Japan; ^5^ Translational Research and Development Center Chiba University Hospital Chiba Japan; ^6^ National Center for Geriatrics and Gerontology Innovation Center for Translational Research Aichi Japan; ^7^ Collaborative Research Laboratory of Medical Innovation, Graduate School of Biomedical and Health Sciences Hiroshima University Hiroshima Japan; ^8^ Research Center for Hepatology and Gastroenterology, Graduate School of Biomedical and Health Sciences Hiroshima University Hiroshima Japan; ^9^ RIKEN Center for Integrative Medical Sciences Yokohama Japan

**Keywords:** BCLC stage‐B, hepatocellular carcinoma, skeletal muscle, sorafenib

## Abstract

**Aim:**

Skeletal muscle volume has been reported to be an important factor that determines overall survival (OS) and post‐progression survival (PPS) in patients with hepatocellular carcinoma (HCC). However, the impact of skeletal muscle volume on HCC with Barcelona Clinic Liver Cancer (BCLC) stage B (BCLC‐B) remains unclear. We conducted sub‐analyses of a previous study on BCLC‐B and compared our findings with data on HCC with BCLC stage C (BCLC‐C).

**Methods:**

We retrospectively enrolled 356 patients with HCC (BCLC‐B, *n* = 78; and BCLC‐C, *n* = 278) undergoing sorafenib therapy. Prognostic factors were analyzed using various parameters, including skeletal muscle volume. Muscle volume (MV) depletion was designated as less than the median value of the skeletal muscle index for each gender (cutoff value: 45.0 cm^2^/m^2^ for male and 38.0 cm^2^/m^2^ for female participants).

**Results:**

Both OS and PPS showed no significant differences in patients with non‐MV depletion and those with MV depletion in the BCLC‐B group (Median OS [MST] 19.3 vs. 13.5 months [*p* = 0.348]; median PPS 9.7 vs. 10.8 months [*p* = 0.578]). In the BCLC‐C group, patients with non‐MV depletion had a significantly longer OS and PPS compared to patients with MV depletion (MST 12.4 vs. 9.0 months [*p* = 0.001] and median PPS 7.9 vs. 5.4 months [*p* = 0.002]). Multivariate analysis revealed that MV depletion was an independent prognostic factor of OS and PPS in the BCLC‐C group but not in the BCLC‐B group.

**Conclusions:**

Skeletal muscle volume showed little impact on the clinical outcomes of patients with BCLC‐B undergoing sorafenib therapy.

## Graphical abstract

Skeletal muscle volume is an important prognostic factor in treating HCC with sorafenib. However, the impact of skeletal muscle volume on HCC with Barcelona Clinic Liver Cancer (BCLC) stage B (BCLC‐B) undergoing sorafenib is unclear. This study showed that muscle volume depletion was an independent prognostic factor of overall survival in the BCLC‐C group but not in the BCLC‐B group. Skeletal muscle volume showed little impact on the clinical outcomes of patients with BCLC‐B undergoing sorafenib therapy.

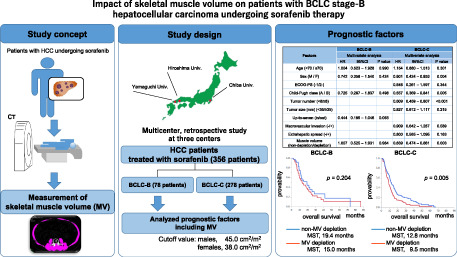

## 1 INTRODUCTION

The new era of molecular targeted agent (MTA) treatment for hepatocellular carcinoma (HCC) has led to the advent of sorafenib.^1^, ^2^ Furthermore, the recent success of combined immunotherapy with atezolizumab and bevacizumab has rapidly renewed the treatment algorithm for advanced HCC. Currently, this combined immunotherapy is being used as a first‐line treatment, and sorafenib is now considered a second‐line agent.^3^, ^4^ A recent retrospective study demonstrated that the second‐line agent with sorafenib and lenvatinib, after the failure of first‐line atezolizumab plus bevacizumab for HCC with Barcelona Clinic Liver Cancer stage C (BCLC‐C), showed equal survival rates.^5^ Therefore, subsequent MTA therapy is required to prolong patient survival. In addition, treatment for HCC with BCLC stage B (BCLC‐B) having refractory transarterial chemoembolization (TACE) or high tumor burden is currently being replaced by MTAs.^3^

Previous studies have shown that skeletal muscle volume is a prognostic factor in treating HCC with sorafenib or lenvatinib.^6^, ^7^, ^8^, ^9^, ^10^ We also reported in a previous study that muscle volume depletion strongly affects overall survival (OS) and post‐progression survival (PPS) in patients with HCC undergoing sorafenib therapy.^11^ However, the impact of skeletal muscle volume on patients with BCLC‐B receiving MTAs is unclear. Furthermore, we found no study in the literature on the topic. We conducted sub‐analyses of a previous study and compared data of BCLC‐B with those of BCLC‐C to resolve this issue.

## 2 METHODS

### 2.1 Study population

This study was a multicenter, retrospective study at three centers in Japan from April 2009 to July 2016. The three centers were the Yamaguchi University Hospital, Chiba University Hospital, and Hiroshima University Hospital. The approval of the study protocol was obtained from each Institutional Ethics Committee (approval numbers: H30‐042, 3253, and E‐1382, respectively). This study was conducted in accordance with the guidelines of the Declaration of Helsinki.

### 2.2 Patients

Data from 356 patients undergoing treatment with sorafenib for HCC in a previous report^11^ were re‐analyzed. We defined the etiology of background liver disease based on positive hepatitis B antigen and/or hepatitis C antibody. According to the BCLC staging system,^12^ there were 78 (21.9%) and 278 (78.1%) patients with BCLC‐B and BCLC‐C, respectively. Of the 356 patients with HCC, 36 (10.1%) did not develop progressive disease (PD) on imaging during the observation period. Therefore, we analyzed PPS in 320 patients, including 69 (21.6%) with BCLC‐B and 251 (78.4%) patients with BCLC‐C (Figure S1).

### 2.3 Treatment

In principle, 800 mg/day of sorafenib was administered initially; however, the dose was reduced according to the patient's condition. Sorafenib was administered until tumor progression or until unacceptable adverse events were observed. Treatment after sorafenib was introduced based on the individual patient's situation.

### 2.4 Assessment of skeletal muscle volume

We measured skeletal muscle volume with computed tomography (CT) imaging of the third lumbar vertebra level using an AZE Virtual Place (Canon Medical Systems Ltd., Tochigi, Japan). To calculate the skeletal muscle concentration, the CT values were set from −29 to +150 Hounsfield units.^13^ We used the following formula (example shown in Figure S2):

### 2.5 Skeletal muscle index (SMI) = skeletal muscle area [cm^2^]/height^2^ [m^2^]

Non‐muscle volume depletion (non‐MV depletion) and muscle volume depletion (MV depletion) were set as cutoff values using the median SMI.

### 2.6 Treatment response evaluation

The radiological images were evaluated every 2–3 months, and the treatment response was classified using the RECIST version 1.1.^14^ The radiological evaluation was performed by each institution, and the best response was defined as the treatment response.

### 2.7 Statistical analysis

All statistical analyses were conducted using JMP Pro version 16 (SAS Institute Inc., Cary, NC, USA), and a *p*‐value less than 0.05 was considered statistically significant. The Fisher's exact or chi‐squared tests were used to compare the groups. The Kaplan–Meier method and log‐rank test were used to estimate the survival time. Prognostic factors were analyzed using Cox proportional hazard regression model. We conducted a follow‐up survey on March 31, 2018, and 273 patients had died before the follow‐up endpoint.

Accordingly, OS and progression‐free survival (PFS) were analyzed in 356 patients. Furthermore, we added “disease control” with sorafenib and “treatment after sorafenib” to the aforementioned factors and analyzed the prognostic factors of PPS in 320 patients who failed sorafenib therapy.

## 3 RESULTS

### 3.1 Baseline patient characteristics

The median SMI in men and women was 45.3 and 38.3 cm^2^/m^2^, respectively (Table 1). Therefore, the cutoff value of MV depletion was designated as 45.0 cm^2^/m^2^ for males and 38.0 cm^2^/m^2^ for females.

**TABLE 1:** Characteristics of patients with Barcelona Clinic Liver Cancer stage B (BCLC‐B) and BCLC‐C.

	Total (*N* = 356)	BCLC‐B	BCLC‐C
Age	69.5 (63.0–75.0)	71.0 (64.8–75.0)	69.0 (62.0–75.0)
Sex (M/F)	287/69	60/18	227/51
Etiology (C/B/B+C/N)	175/80/2/99	44/11/0/23	131/69/2/76
ECOG‐PS 0/1/2/3	314/37/3/2	78/0/0/0	237/36/3/2
Child–Pugh class (A/B)	310/46	70/8	240/38
Tumor number	8 (2–8)	8 (4–8)	8 (2–8)
Tumor size (mm)	35.0 (18.3–65.0)	28 (17–49.3)	38.5 (19.0–70.3)
Up‐to‐seven (in/out)	92/262	10/68	82/194
Macrovascular invasion (−/+)	258/98	78/0	184/94
Extrahepatic spread (−/+)	167/189	78/0	186/92
SMI			
M	45.3 (41.2–50.4)	45.3 (41.6–51.7)	45.3 (41.1–50.1)
F	38.3 (34.0–42.9)	34.6 (31.7–43.2)	38.8 (34.6–43.0)
Muscle volume (non‐depletion/depletion)	181/175	37/41	144/134

Abbreviations: B, hepatitis B virus; BCLC, Barcelona Clinic Liver Cancer; C, hepatitis C virus; ECOG‐PS, Eastern Cooperative Oncology Group performance status; EHS, extrahepatic spread; F, female; M, male; MVI, macrovascular invasion; N, NonBnonC; SMI, skeletal muscle index.

In the BCLC‐B group (*n* = 78), the median age was 71.0, and 60 (76.9%) were men. Regarding liver function, 70 (89.7%) and eight patients (10.3%) had Child–Pugh A and B diseases, respectively. MV depletion was observed in 41 patients (52.6%).

In the BCLC‐C group (*n* = 278), the median age was 69.0, and 227 (81.7%) were men. Regarding liver function, 240 (86.3%) and 38 patients (13.7%) had Child–Pugh A and B disease, respectively. MV depletion was observed in 134 patients (48.2%). The clinical characteristics used in the analysis of PPS are shown in Tables S1 and S2.

### 3.2 Treatment response

The objective response rate (ORR) and disease control rate (DCR) in the BCLC‐B group were 5.1% and 69.2%, respectively: complete response [CR]/partial response [PR]/stable disease [SD]/progressive disease [PD] = 0 (0%)/4 (5.1%)/50 (64.1%)/24 (30.8%). Alternatively, ORR and DCR in the BCLC‐C group were 4.3% and 57.2%, respectively: CR/PR/SD/PD = 0 (0%)/12 (4.3%)/147 (52.9%)/119 (42.8%). Therefore, the overall ORR and DCR for all the patients were 4.5% and 59.8%, respectively.

### 3.3 OS, PPS, and PFS in patients with BCLC‐B

**FIGURE 1:**
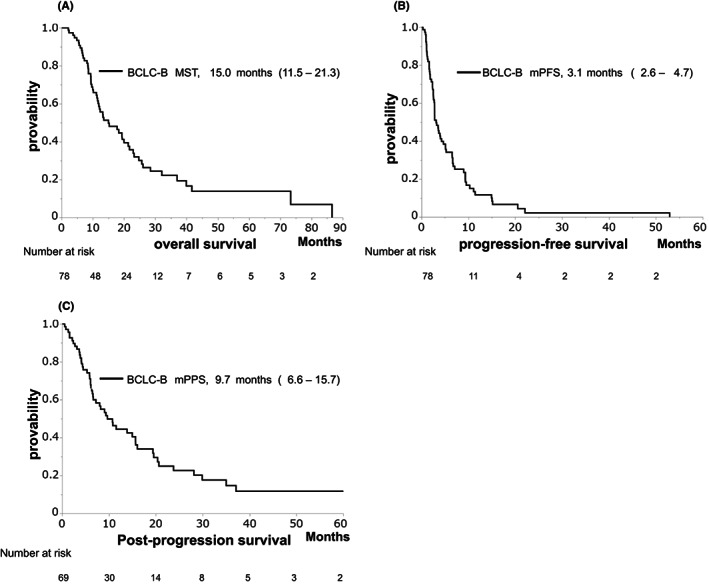
Kaplan–Meier analysis of survival; overall survival (OS) (A), progression‐free survival (PFS) (B), and post‐progression survival (PPS) (C) in the Barcelona Clinic Liver Cancer stage B (BCLC‐B) group.

**FIGURE 2:**
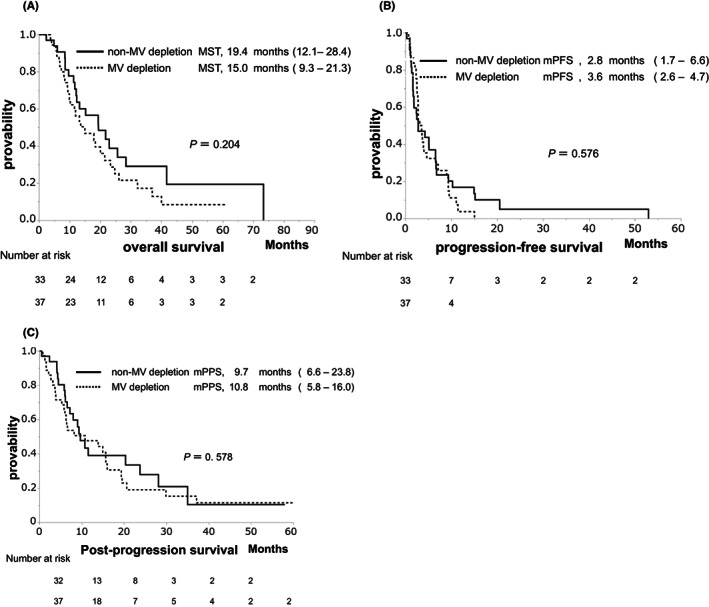
Cumulative survival according to the skeletal muscle volume in the Barcelona Clinic Liver Cancer stage B (BCLC‐B) group. (A) Overall survival between the non‐muscle volume (non‐MV) depletion and muscle volume (MV) depletion groups (MST: 19.3 vs. 15.0 months, *p* = 0.348). (B) Progression‐free survival (PFS) between the non‐MV depletion and MV depletion groups (median PFS: 2.8 vs. 3.2 months, *p* = 0.638). (C) Post‐progression survival (PPS) between the non‐MV depletion and MV depletion groups (median PPS: 9.7 vs. 10.8 months, *p* = 0.578).

The median OS (MST), PFS, and PPS were 15.0, 3.1, and 9.7 months, respectively (Figure 1A–C). These clinical outcomes demonstrated no significant differences between the non‐MV depletion and MV depletion groups (MST: 19.3 vs. 13.5 months, *p* = 0.348, Figure 2A; median PFS: 2.8 vs. 3.2 months, *p* = 0.638, Figure 2B; median PPS: 9.7 vs. 10.8 months, *p* = 0.578, Figure 2C).

### 3.4 OS, PPS, and PFS in patients with BCLC‐C

**FIGURE 3:**
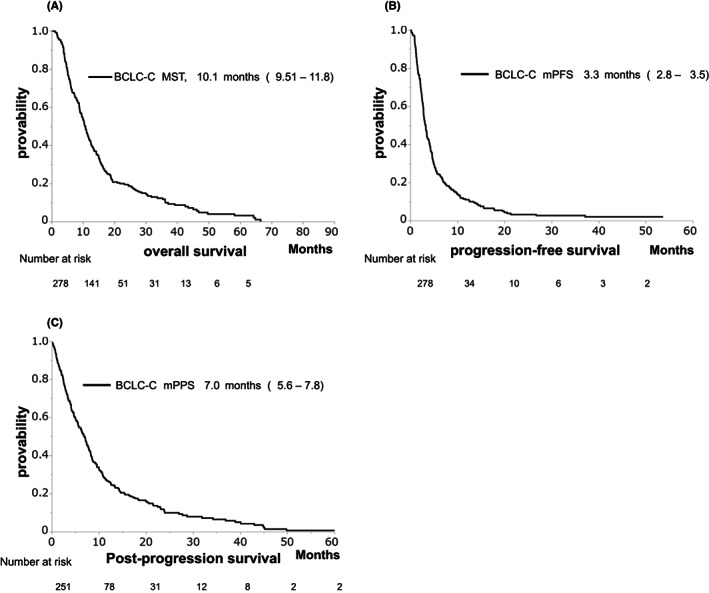
Kaplan–Meier analysis of survival: overall survival (OS) (A), progression‐free survival (PFS) (B), and post‐progression survival (PPS) (C) in the Barcelona Clinic Liver Cancer stage C (BCLC‐C) group.

**FIGURE 4:**
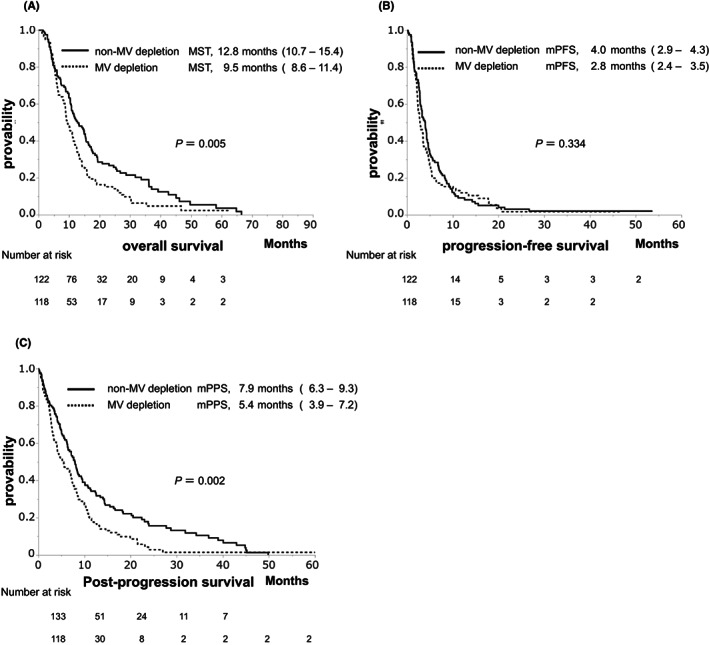
Cumulative survival according to skeletal muscle volume in the Barcelona Clinic Liver Cancer stage C (BCLC‐C) group. (A) Overall survival between the non‐muscle volume (non‐MV) depletion and muscle volume (MV) depletion groups (MST: 12.4 vs. 9.0 months, *p* = 0.001). (B) Progression‐free survival (PFS) between the non‐MV depletion and MV depletion groups (median PFS: 3.6 vs. 2.9 months, *p* = 0.346). (C) Post‐progression survival (PPS) between the non‐MV depletion and MV depletion groups (MST: 7.9 vs. 5.4 months, *p* = 0.002).

The MST, median PFS, and PPS were 10.1, 3.3, and 7.0 months, respectively (Figure 3A–C). The non‐MV depletion group experienced significantly longer OS compared to the MV depletion group (MST: 12.4 vs. 9.0 months, *p* = 0.010, Figure 4A). However, there was no significant difference in PFS between the non‐MV depletion and MV depletion groups (median PFS: 3.6 vs. 2.9 months, *p* = 0.346, Figure 4B). The non‐MV depletion group experienced significantly longer PPS compared to the MV depletion group (MST: 7.9 vs. 5.4 months, *p* = 0.002, Figure 4C).

### 3.5 Prognostic factors of PPS or OS

As shown in Table 2, no significant prognostic factors of PPS were demonstrated in the BCLC‐B group. In contrast, in the BCLC‐C group, the multivariate analysis depicted four prognostic factors of PPS: male sex (hazard ratio [HR]: 0.601, *p* = 0.004), Child–Pugh A (HR: 0.557, *p* = 0.005), tumor number <8 (HR: 0.609, *p* < 0.001), and non‐MV depletion (HR: 0.639, *p* = 0.003) (Table 2).

**TABLE 2:** Prognostic factors of post‐progression survival in the Barcelona Clinic Liver Cancer stage B (BCLC‐B) and BCLC‐C groups.

Factors	BCLC‐B				BCLC‐C			
	Univariate analysis	Multivariate analysis			Univariate analysis	Multivariate analysis		
	*p* value	HR	95%CI	*p* value	*p* value	HR	95%CI	*p* value
Age (<70/≥70)	0.787	1.004	0.523–1.928	0.990	0.282	1.154	0.880–1.513	0.301
Sex (M/F)	0.520	0.742	0.358–1.540	0.424	0.037	0.601	0.424–0.853	0.004
ECOG‐PS (−1/2‐)					0.378	0.646	0.261–1.597	0.344
Child–Pugh class (A/B)	0.632	0.725	0.287–1.837	0.498	0.011	0.557	0.369–0.841	0.005
Tumor number (<8/≥8)	0.317				<0.001	0.609	0.459–0.807	<0.001
Tumor size (mm) (<35/≥35)	0.746				0.074	0.827	0.612–1.117	0.215
Up‐to‐seven (in/out)	0.132	0.444	0.189–1.046	0.063				
Macrovascular invasion (−/+)					0.006	0.909	0.642–1.287	0.589
Extrahepatic spread (−/+)					0.523	0.800	0.585–1.095	0.163
Muscle volume (non‐depletion/depletion)	0.579	1.007	0.525–1.931	0.984	0.002	0.639	0.474–0.861	0.003

Abbreviations: CI, confidence interval; ECOG‐PS, Eastern Cooperative Oncology Group performance status; EHS, extrahepatic spread; F, female; HR, hazard ratio; M, male; MVI, macrovascular invasion.

Furthermore, there were no significant prognostic factors of OS in the BCLC‐B group (Table 3). However, five factors, including male sex (HR: 0.635, *p* = 0.010), Child–Pugh A (HR: 0.685, *p* = 0.049), tumor number <8 (HR: 0.540, *p* < 0.001), extra‐hepatic spread (EHS) (−) (HR: 0.676, *p* = 0.011), and non‐MV depletion (HR: 0.650, *p* = 0.002), were significant prognostic factors of OS following multivariate analysis of the BCLC‐C group (Table 3).

**TABLE 3:** Prognostic factors of overall survival in the Barcelona Clinic Liver Cancer stage B (BCLC‐B) and BCLC‐C groups.

Factors	BCLC‐B				BCLC‐C			
	Univariate analysis	Multivariate analysis			Univariate analysis	Multivariate analysis		
	*p* value	HR	95%CI	*p* value	*p* value	HR	95%CI	*p* value
Age (<70/≥70)	0.335	0.844	0.464–1.535	0.578	0.389	1.082	0.836–1.402	0.549
Sex (M/F)	0.174	0.609	0.315–1.177	0.140	0.143	0.635	0.451–0.895	0.010
ECOG‐PS (−1/2‐)					0.333	0.619	0.251–1.527	0.298
Child–Pugh class (A/B)	0.324	0.725	0.287–1.837	0.498	0.045	0.685	0.470–0.999	0.049
Tumor number (<8/≥8)	0.389				<0.001	0.540	0.412–0.708	<0.001
Tumor size (mm) (<35/≥35)	0.949				0.068	0.885	0.664–1.180	0.405
Up‐to‐seven (in/out)	0.050	0.444	0.189–1.046	0.063				
Macrovascular invasion (−/+)					0.001	0.780	0.561–1.086	0.141
Extrahepatic spread (−/+)					0.688	0.676	0.499–0.914	0.011
Muscle volume (non‐depletion/depletion)	0.350	1.054	0.580–1.916	0.862	0.002	0.650	0.492–0.858	0.002

Abbreviations: CI, confidence interval; ECOG‐PS, Eastern Cooperative Oncology Group performance status; EHS, extrahepatic spread; F, female; HR, hazard ratio; M, male; MVI, macrovascular invasion.

### 3.6 Prognostic factors of OS in patients with progressive disease

In the BCLC‐B group, two significant prognostic factors for OS were extracted by the multivariate analysis: disease control (yes) (HR: 0.370, *p* = 0.005) and treatment after sorafenib (yes) (HR: 0.427, *p* = 0.019) (Table 4).

**TABLE 4:** Prognostic factors of overall survival in the Barcelona Clinic Liver Cancer stage B (BCLC‐B) and BCLC‐C patients with disease progression.

Factors	BCLC‐B				BCLC‐C			
	Univariate analysis	Multivariate analysis			Univariate analysis	Multivariate analysis		
	*p* value	HR	95%CI	*p* value	*p* value	HR	95%CI	*p* value
Age (<70/≥70)	0.598	1.064	0.567–2.000	0.846	0.350	1.066	0.809–1.404	0.651
Sex (M/F)	0.307	1.049	0.465–2.367	0.908	0.049	0.678	0.475–0.968	0.033
ECOG‐PS (−1/2‐)					0.323	0.609	0.242–1.531	0.292
Child–Pugh class (A/B)	0.696	1.550	0.432–5.558	0.501	0.050	0.582	0.387–0.874	0.009
Tumor number (<8/≥8)	0.411				<0.001	0.561	0.422–0.747	<0.001
Tumor size (mm) (<35/≥35)	0.896				0.054	0.778	0.568–1.065	0.117
Up‐to‐seven (in/out)	0.083	0.802	0.292–2.201	0.669				
Macrovascular invasion (−/+)					0.002	1.023	0.717–1.459	0.901
Extrahepatic spread (−/+)					0.904	0.777	0.569–1.059	0.111
Muscle volume (non‐depletion/depletion)	0.417	0.720	0.368–1.408	0.338	<0.001	0.508	0.371–0.695	<0.001
Disease control (yes/no)	0.005	0.370	0.183–0.745	0.005	<0.001	0.414	0.311–0.550	<0.001
Treatment after soafenib (yes/no)	0.005	0.427	0.210–0.868	0.019	0.003	0.573	0.429–0.764	<0.001

Abbreviations: CI, confidence interval; ECOG‐PS, Eastern Cooperative Oncology Group performance status; EHS, extrahepatic spread; F, female; HR, hazard ratio: M, male; MVI, macrovascular invasion.

In the BCLC‐C group, the following six factors were significant prognostic factors following the multivariate analysis: male sex (HR: 0.678, *p* = 0.033), Child–Pugh A (HR: 0.582, *p* = 0.009), tumor number <8 (HR: 0.561, *p* < 0.001), non‐MV depletion (HR: 0.508, *p* < 0.001), disease control (yes) (HR: 0.414, *p* < 0.001), and treatment after sorafenib (yes) (HR: 0.573, *p* < 0.001) (Table 4).

## 4 DISCUSSION

The appearance of several MTAs and immunotherapy is leading to a paradigm shift in systemic therapy for HCC.^3^, ^4^ Previously, the primary treatment of BCLC‐B HCC has been understood to be TACE in several guidelines.^15^, ^16^, ^17^, ^18^ However, retrospective and prospective studies have shown that TACE‐refractory patients that were switched to sorafenib demonstrated more prolonged survival than TACE‐refractory patients who received repetitive TACE treatment.^19^, ^20^ Currently, MTAs have widely been used for patients with BCLC‐B HCC.

There are various reports about the survival impact of MV depletion in patients with HCC treated with MTAs.^6^, ^7^, ^8^, ^9^, ^10^, ^11^ However, as patients with all stages of HCC were analyzed in these studies, the impact of MV depletion on patients with BCLC‐B who received MTAs remains undetermined. Thus, we re‐evaluated whether skeletal muscle volume was associated with clinical outcomes in patients with BCLC‐B undergoing sorafenib therapy using previous data. OS, PFS, and PPS showed similar curves between the non‐MV depletion and MV depletion groups among patients with BCLC‐B. Moreover, MV depletion was not a prognostic factor of PPS or OS.

In contrast, patients with non‐MV depletion demonstrated significantly longer PPS and OS than those with MV depletion in the BCLC‐C group, and MV depletion was a poor prognostic factor of PPS or OS. Therefore, these findings indicate different impacts of skeletal muscle volume on patient clinical outcomes between those with BCLC‐B and those with BCLC‐C. Additionally, other reports have shown that baseline skeletal muscle volume did not affect survival in patients with HCC who received TACE and/or transarterial infusion. However, a rapid decrease in skeletal muscle was a poor prognostic factor.^21^, ^22^ Although we were unable to analyze the change in skeletal muscles during sorafenib treatment in this study, another report has demonstrated that patients with HCC undergoing sorafenib treatment who experienced a rapid decrease of skeletal muscle volume showed a poor prognosis.^23^ We previously demonstrated that sorafenib treatment for HCC tended to rapidly decrease skeletal muscle mass compared to hepatic arterial infusion chemotherapy (HAIC) (7.2% vs. 2.7% at 3 months after therapy, *p* = 0.095).^24^ Furthermore, most patients with intermediate‐stage HCC progress to advanced‐stage HCC; therefore, the management of the skeletal muscle volume with nutritional therapies and cancer rehabilitation^25^ will be required.

Regarding prognostic factors, OS, disease control, and treatment after sorafenib therapy were extracted independently through multivariate analysis in patients with BCLC‐B. Interestingly, the tumor number was not extracted; thus, it has been demonstrated that patients with BCLC‐B with beyond the up‐to‐seven criteria will be good candidates for sorafenib therapy.

This study had certain limitations. The number of patients with BCLC‐B was small; however, a multicenter study may reduce patient selection bias among the respective centers. Furthermore, HCC patients with Child–Pugh A function are usually scheduled for sorafenib treatment. We performed sub‐analysis only in the patients with Child–Pugh A and confirmed similar findings (data not shown). In real‐world clinical practice, patients with Child–Pugh B function often receive sorafenib. We adopted the findings in the cohort, including patients with Child–Pugh B. Next, we used median SMI values in this study. The criteria for muscle depletion were SMI <36.2–52.4 cm^2^/m^2^ for males and SMI <29.0–39.5 cm^2^/m^2^ for females.^26^ The Japan Society of Hepatology established that the criteria for skeletal muscle depletion for SMI were <42 cm^2^/m^2^ for males and <38 cm^2^/m^2^ for females.^27^ Even when the cutoff values of these criteria were used in this study, OS demonstrated no significant differences between patients with (*n* = 29) and without (*n* = 49) muscle depletion in the BCLC‐B group (MST: 13.1 vs. 19.3 months, *p* = 0.744) (Figure S3a). In the BCLC‐C group, those without muscle depletion (*n* = 88) tended to have a longer OS than those with muscle depletion (*n* = 90) (MST: 11.2 vs. 9.0 months, *p* = 0.085) (Figure S3b). We examined another cutoff value of the SMI using a time‐dependent receiver operating characteristic analysis and Harrell's c‐index for men and women according to the model of the report produced by Heagerty et al.^28^ Consequently, 45 cm^2^/m^2^ for men (same as the median value) and 40 cm^2^/m^2^ for women were calculated as each optimal cutoff value. The clinical outcomes of OS in the BCLC‐B group were the same results because no female patients with SMI less than 40 cm^2^/m^2^ from 38 cm^2^/m^2^ were found, and those in the BCLC‐C group showed similar results (data not shown). Therefore, even if several cutoff values of SMI were used, skeletal muscle volume showed little impact on the clinical outcomes of BCLC‐B compared with BCLC‐C. Finally, the enrollment period of this study was between April 2009 and July 2016, and the follow‐up endpoint was March 2018. As regorafenib could only be used during this period, the recent treatment trend after sorafenib was markedly different. However, initiating treatment after sorafenib, such as TACE and HAIC, was a prognostic factor in both BCLC‐C and BCLC‐B groups in this study. In the new era of MTAs and immunotherapy, sequential therapy may improve survival, as demonstrated by the results of our study.

In conclusion, our sub‐analyses of this retrospective multicenter study revealed that MV depletion was not associated with clinical outcomes in patients with BCLC‐B HCC undergoing sorafenib therapy. However, even such patients will need to be prepared for the preservation or upregulation of skeletal muscle volume before entering advanced stages.

## AUTHOR CONTRIBUTIONS

**Issei Saeki:** Conceptualization (equal); data curation (equal); formal analysis (equal); writing – original draft (equal). **Takahiro Yamasaki:** Conceptualization (equal); investigation (equal); writing – original draft (equal). **Yurika Yamauchi:** Data curation (equal). **Tomokazu Kawaoka:** Data curation (equal). **shinsuke Uchikawa:** Data curation (equal). **Akira Hiramatsu:** Data curation (equal). **Hiroshi Aikata:** Data curation (equal). **Kazufumi Kobayashi:** Data curation (equal). **Takayuki Kondo:** Data curation (equal). **Sadahisa Ogasawara:** Data curation (equal). **Tetsuhiro Chiba:** Data curation (equal). **Reo Kawano:** Formal analysis (equal). **Kazuaki Chayama:** Writing – review and editing (equal). **Naoya Kato:** Writing – review and editing (equal). **Taro Takami:** Writing – review and editing (equal).

## FUNDING INFORMATION

This study received no financial support.

## CONFLICT OF INTEREST STATEMENT

Hiroshi Aikata: lecture fees from Bayer and Eisai.Sadahisa Ogasawara: lecture fees and research funding from Bayer and Eisai.Tetsuhiro Chiba: lecture fees from Eisai.Kazuaki Chayama: lecture fees from AbbVie, Bristol Myers Squibb, Gilead Sciences Inc, MSD, Otsuka, and Sumitomo Dainippon Pharma, Tanabe Mitsubishi, as well as research funding from Sumitomo Dainippon Pharma.Naoya Kato: lecture fees from Bayer, Chugai, Eisai, and Takeda, and research funding from Bayer and Eisai.The remaining authors have no conflicts of interest.

## ETHICS STATEMENT

This present study was conducted in conformity with the guidelines of the Declaration of Helsinki, and this protocol obtained approval from the Institutional Ethics Committee (H30‐042 [Yamaguchi University Hospital], 3253 [Chiba University Hospital], E‐1382 [Hiroshima University Hospital]).

## PATIENT CONSENT STATEMENT

This is a multicenter study with a retrospective analysis using existing data. Patient consent for the disclosure of this study, with an opt‐out option, was obtained.

## Supporting information


**Supplementary Figure 1.** Study chart.
**Supplementary Figure 2.** Measurement of skeletal muscle index (SMI).
The patient was a 61‐year old man. He was 1.61 m in height and weighed 77.5 kg. The skeletal muscle area (SMA) with computed tomography (CT) of the third lumbar vertebral level was analyzed using an AZE virtual place (SMA, pink‐colored area). The SMI was calculated from SMA divided by the square of height; 141.8 [cm^2^]/1.61[m]^2^ = 54.6.
**Supplementary Figure 3.** Kaplan–Meier analysis of overall survival (OS) based on the values of skeletal muscle mass index (SMI) using the Japan Society of Hepatology (JSH) criteria. The cutoff SMI values were 42 cm^2^/m^2^ for males and 38 cm^2^/m^2^ for females.
**a.** OS between Barcelona Clinic Liver Cancer stage B (BCLC‐B) with and without muscle depletion (median OS [mOS]: 19.3 vs. 13.1 months, *p* = 0.744).
**b.** OS between Barcelona Clinic Liver Cancer stage C (BCLC‐C) with and without muscle depletion (mOS: 11.2 vs. 9.0 months, *p* = 0.085).
**Supplementary Table 1.** Characteristics of patients with Barcelona Clinic Liver Cancer stage B (BCLC‐B) according to the skeletal muscle volume
**Supplementary Table 2.** Characteristics of patients with Barcelona Clinic Liver Cancer stage C (BCLC‐C) according to the skeletal muscle volume

## Data Availability

This study is a multicenter retrospective study. We obtained anonymous data from each hospitals for the specialized purpose in this study. Therefore, the dataset of this study is not shown publicly.
