# Supplementary material for: Impact of skeletal muscle volume on patients with BCLC stage‐B hepatocellular carcinoma undergoing sorafenib therapy
Source: Cancer Med. 2023 Mar 23;12(9):10625–35. doi: 10.1002/cam4.5810 (PMC10225214; doi:10.1002/cam4.5810)
Supplement: Supplementary file 1 — Supplementary Figure 1. Study chart. Supplementary Figure 2. Measurement of skeletal muscle index (SMI). The patient was a 61‐year old man. He was 1.61 m in height and weighed 77.5 kg. The skeletal muscle area (SMA) with computed tomography (CT) of the third lumbar vertebral level was analyzed using an AZE virtual place (SMA, pink‐colored area). The SMI was calculated from SMA divided by the square of height; 141.8 [cm2]/1.61[m]2 = 54.6. Supplementary Figure 3. Kaplan–Meier analysis of overall survival (OS) based on the values of skeletal muscle mass index (SMI) using the Japan Society of Hepatology (JSH) criteria. The cutoff SMI values were 42 cm2/m2 for males and 38 cm2/m2 for females. a. OS between Barcelona Clinic Liver Cancer stage B (BCLC‐B) with and without muscle depletion (median OS [mOS]: 19.3 vs. 13.1 months, p = 0.744). b. OS between Barcelona Clinic Liver Cancer stage C (BCLC‐C) with and without muscle depletion (mOS: 11.2 vs. 9.0 months, p = 0.085). Supplementary Table 1. Characteristics of patients with Barcelona Clinic Liver Cancer stage B (BCLC‐B) according to the skeletal muscle volume Supplementary Table 2. Characteristics of patients with Barcelona Clinic Liver Cancer stage C (BCLC‐C) according to the skeletal muscle volume [file CAM4-12-10625-s001.pptx]

## Slide 1
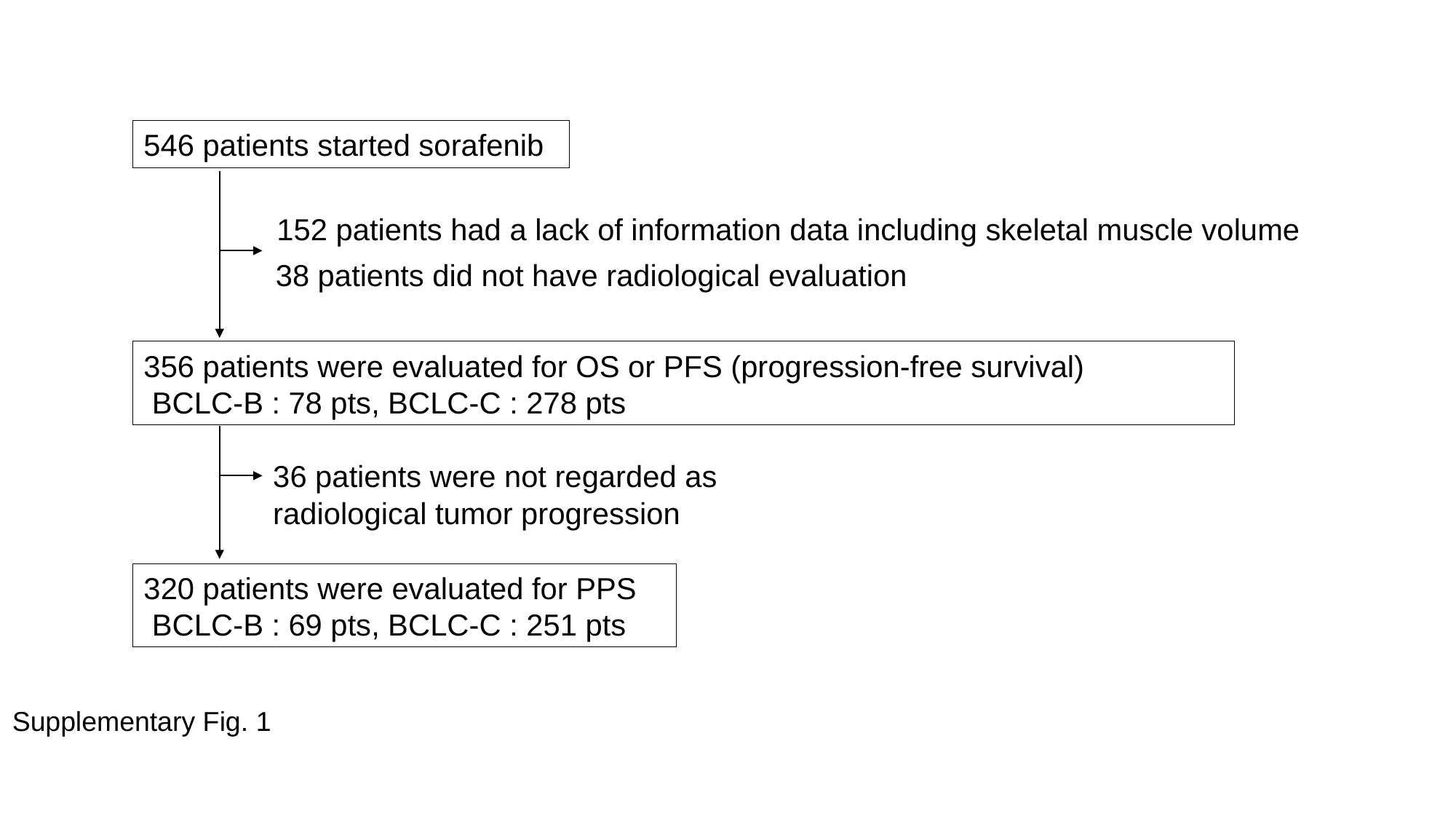

546 patients started sorafenib
152 patients had a lack of information data including skeletal muscle volume
38 patients did not have radiological evaluation
356 patients were evaluated for OS or PFS (progression-free survival)
 BCLC-B : 78 pts, BCLC-C : 278 pts
36 patients were not regarded as radiological tumor progression
320 patients were evaluated for PPS
 BCLC-B : 69 pts, BCLC-C : 251 pts
Supplementary Fig. 1

## Slide 2
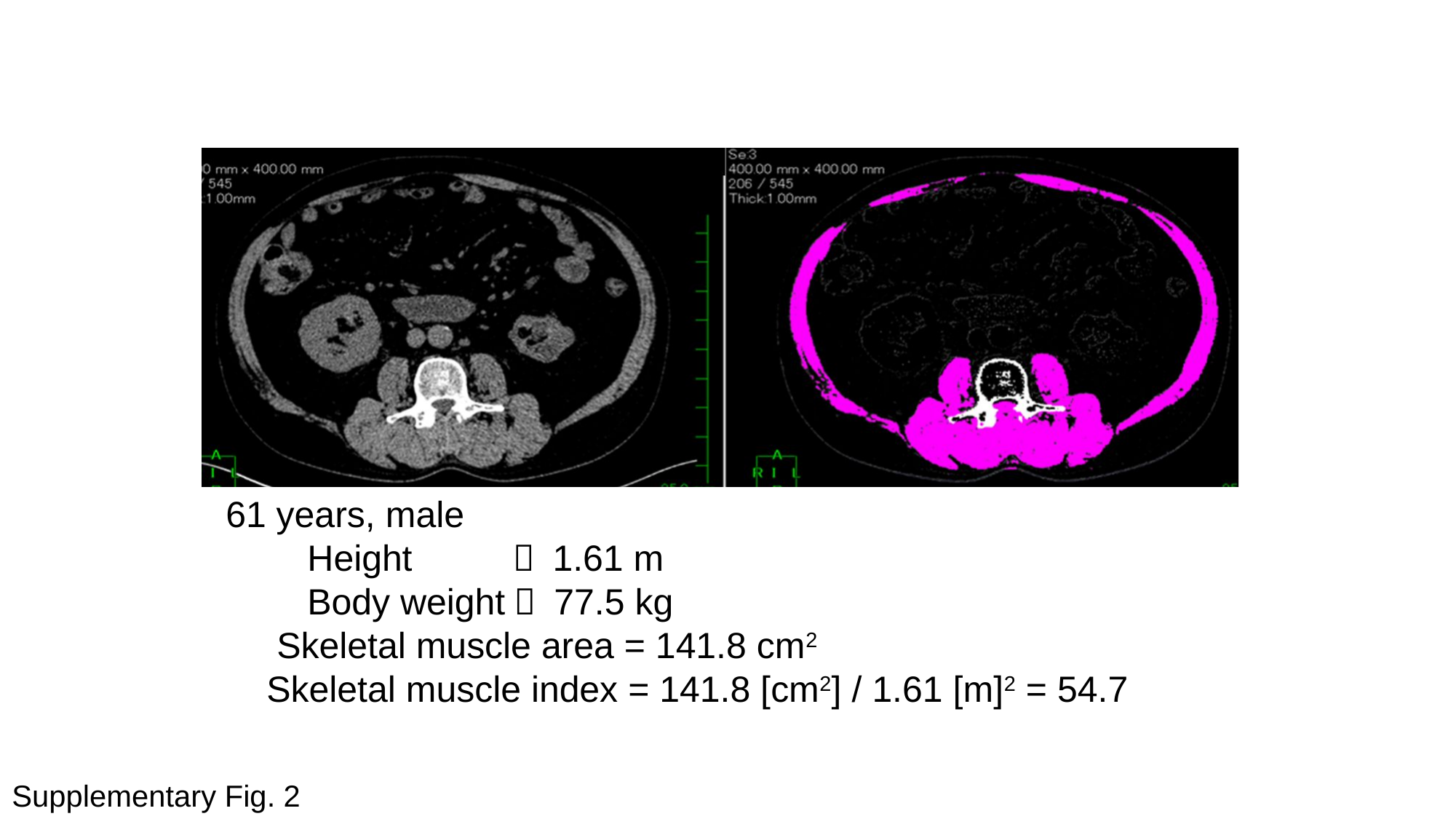

61 years, male
　　Height ： 1.61 m
　　Body weight： 77.5 kg
 Skeletal muscle area = 141.8 cm2
 Skeletal muscle index = 141.8 [cm2] / 1.61 [m]2 = 54.7
Supplementary Fig. 2

## Slide 3
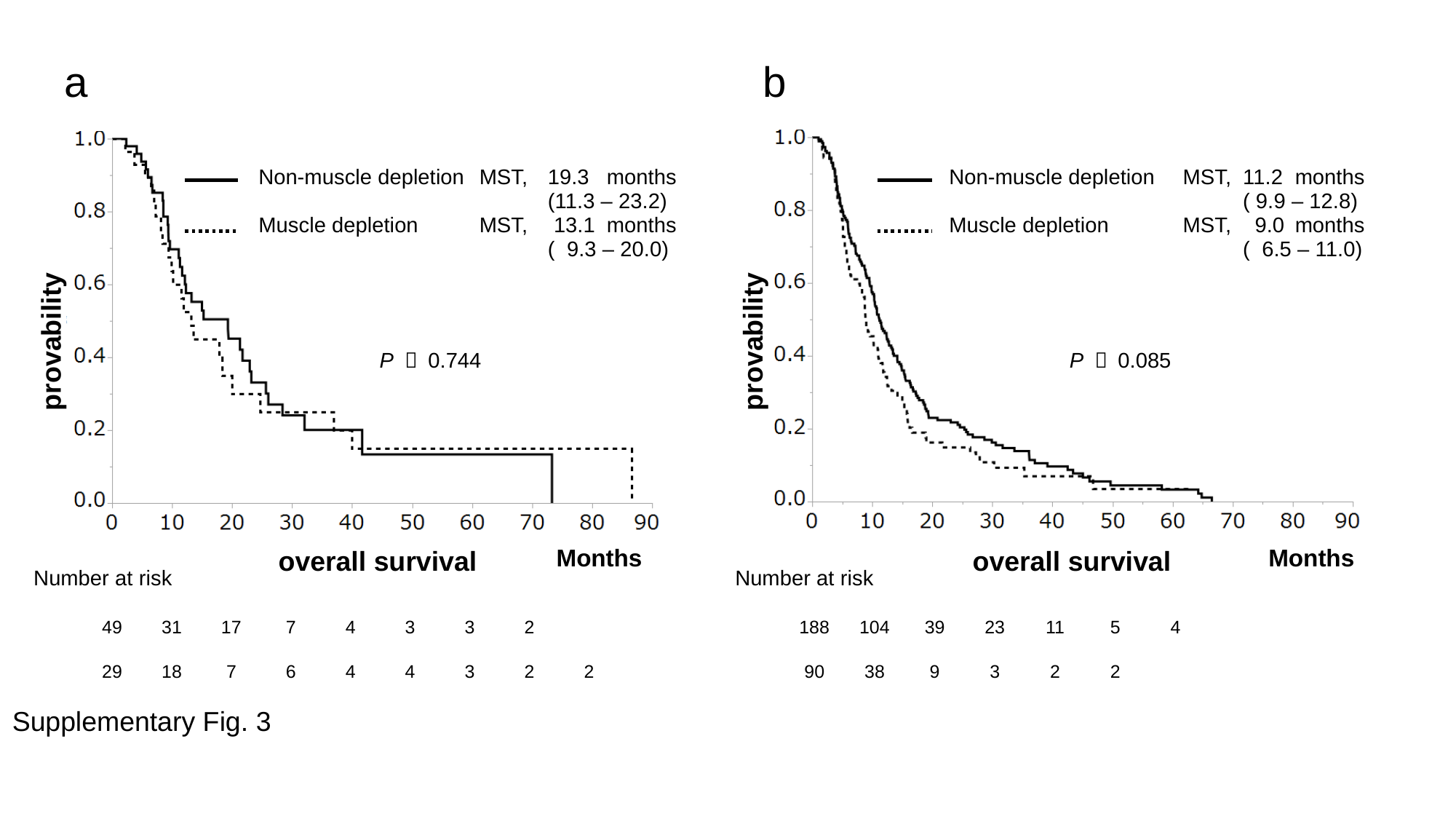

a
b
| | Non-muscle depletion | MST, | 19.3 | months |
| --- | --- | --- | --- | --- |
| | | | (11.3 – 23.2) | |
| | Muscle depletion | MST, | 13.1 | months |
| | | | ( 9.3 – 20.0) | |
| | Non-muscle depletion | MST, | 11.2 | months |
| --- | --- | --- | --- | --- |
| | | | ( 9.9 – 12.8) | |
| | Muscle depletion | MST, | 9.0 | months |
| | | | ( 6.5 – 11.0) | |
provability
provability
P ＝ 0.744
P ＝ 0.085
overall survival
overall survival
Months
Months
| Number at risk | | | | | | | | | | |
| --- | --- | --- | --- | --- | --- | --- | --- | --- | --- | --- |
| | 49 | 31 | 17 | 7 | 4 | 3 | 3 | 2 | | |
| | 29 | 18 | 7 | 6 | 4 | 4 | 3 | 2 | 2 | |
| Number at risk | | | | | | | |
| --- | --- | --- | --- | --- | --- | --- | --- |
| | 188 | 104 | 39 | 23 | 11 | 5 | 4 |
| | 90 | 38 | 9 | 3 | 2 | 2 | |
Supplementary Fig. 3

## Slide 4
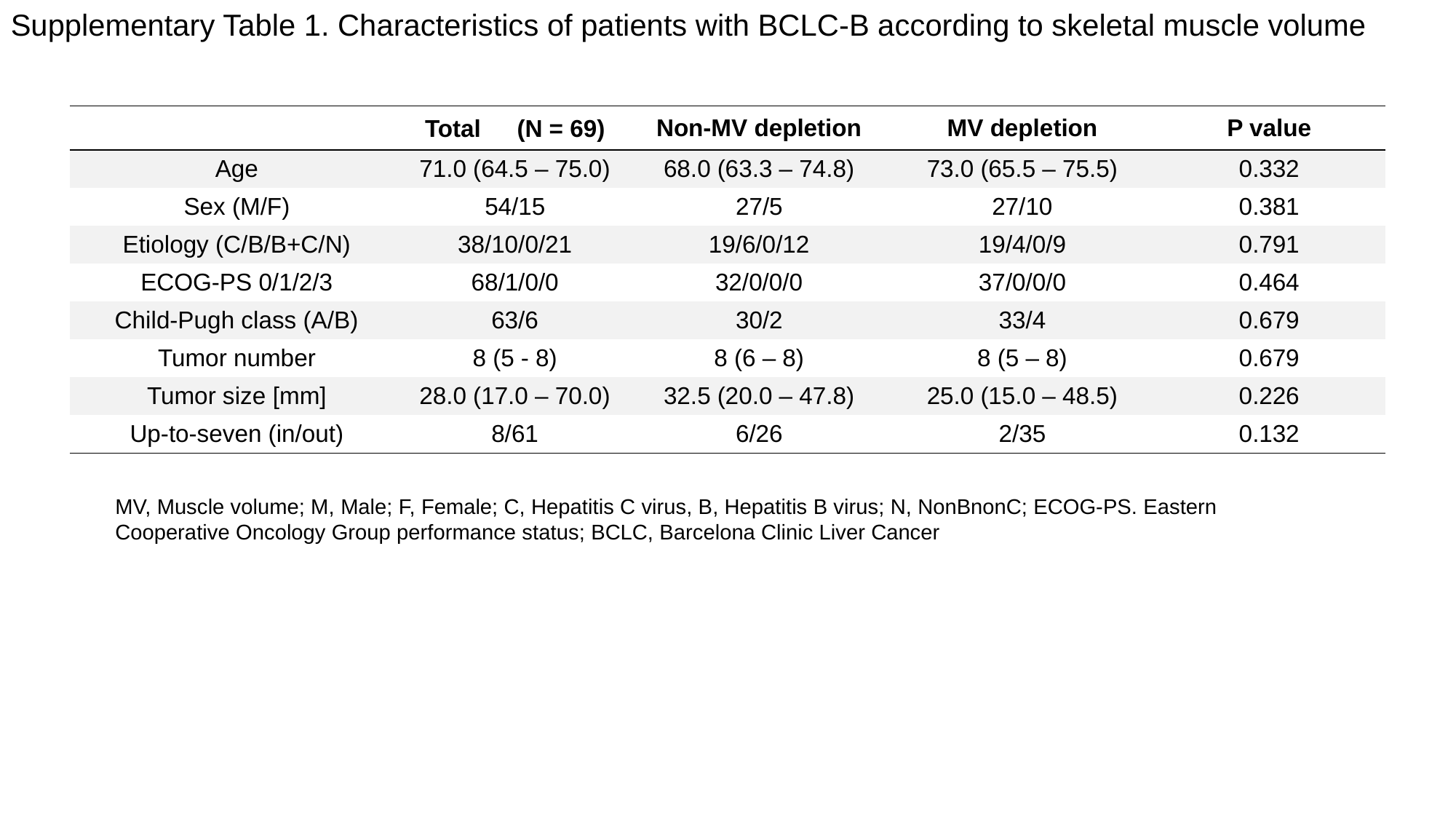

Supplementary Table 1. Characteristics of patients with BCLC-B according to skeletal muscle volume
| | Total　(N = 69) | Non-MV depletion | MV depletion | P value |
| --- | --- | --- | --- | --- |
| Age | 71.0 (64.5 – 75.0) | 68.0 (63.3 – 74.8) | 73.0 (65.5 – 75.5) | 0.332 |
| Sex (M/F) | 54/15 | 27/5 | 27/10 | 0.381 |
| Etiology (C/B/B+C/N) | 38/10/0/21 | 19/6/0/12 | 19/4/0/9 | 0.791 |
| ECOG-PS 0/1/2/3 | 68/1/0/0 | 32/0/0/0 | 37/0/0/0 | 0.464 |
| Child-Pugh class (A/B) | 63/6 | 30/2 | 33/4 | 0.679 |
| Tumor number | 8 (5 - 8) | 8 (6 – 8) | 8 (5 – 8) | 0.679 |
| Tumor size [mm] | 28.0 (17.0 – 70.0) | 32.5 (20.0 – 47.8) | 25.0 (15.0 – 48.5) | 0.226 |
| Up-to-seven (in/out) | 8/61 | 6/26 | 2/35 | 0.132 |
MV, Muscle volume; M, Male; F, Female; C, Hepatitis C virus, B, Hepatitis B virus; N, NonBnonC; ECOG-PS. Eastern Cooperative Oncology Group performance status; BCLC, Barcelona Clinic Liver Cancer

## Slide 5
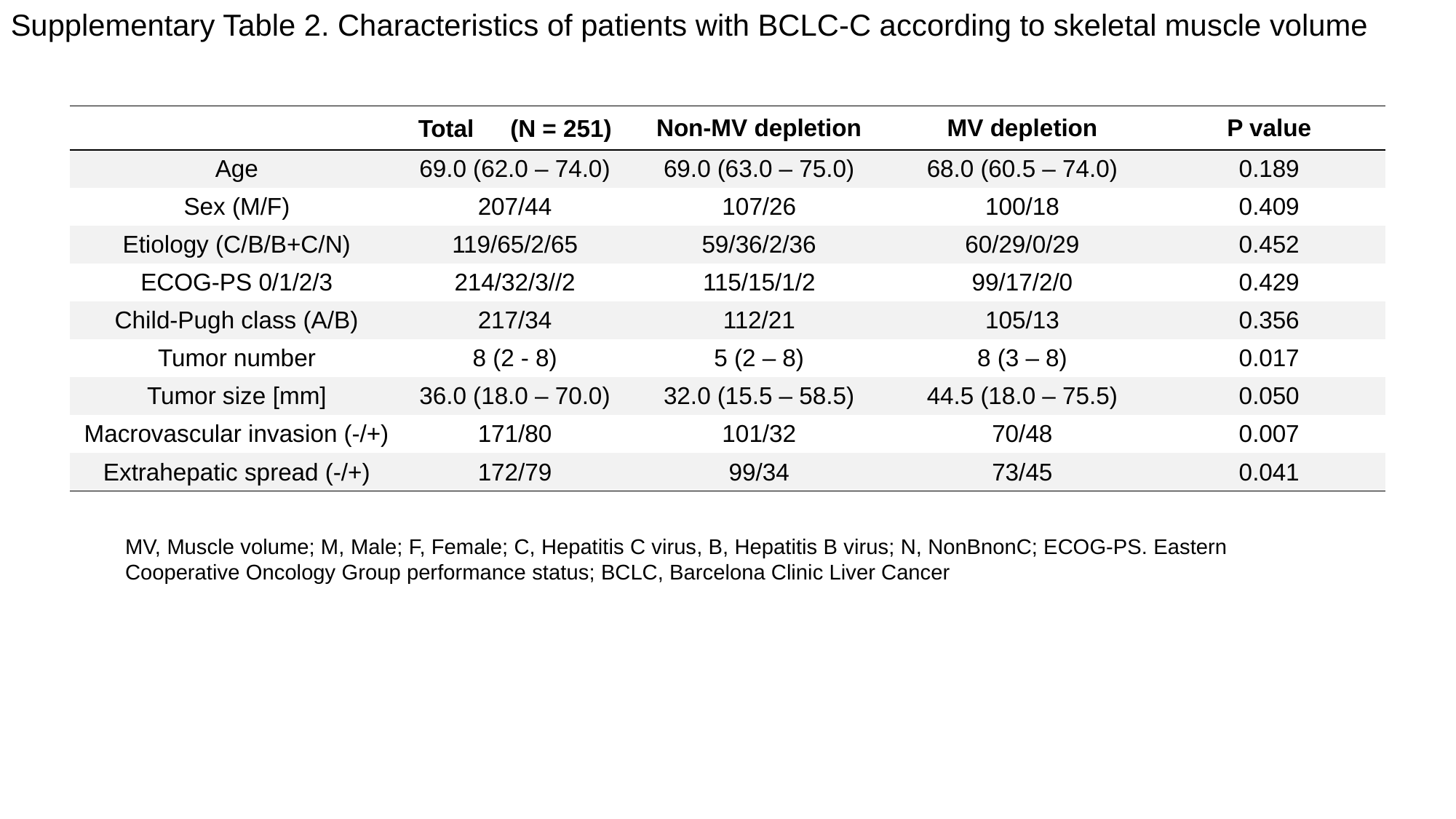

Supplementary Table 2. Characteristics of patients with BCLC-C according to skeletal muscle volume
| | Total　(N = 251) | Non-MV depletion | MV depletion | P value |
| --- | --- | --- | --- | --- |
| Age | 69.0 (62.0 – 74.0) | 69.0 (63.0 – 75.0) | 68.0 (60.5 – 74.0) | 0.189 |
| Sex (M/F) | 207/44 | 107/26 | 100/18 | 0.409 |
| Etiology (C/B/B+C/N) | 119/65/2/65 | 59/36/2/36 | 60/29/0/29 | 0.452 |
| ECOG-PS 0/1/2/3 | 214/32/3//2 | 115/15/1/2 | 99/17/2/0 | 0.429 |
| Child-Pugh class (A/B) | 217/34 | 112/21 | 105/13 | 0.356 |
| Tumor number | 8 (2 - 8) | 5 (2 – 8) | 8 (3 – 8) | 0.017 |
| Tumor size [mm] | 36.0 (18.0 – 70.0) | 32.0 (15.5 – 58.5) | 44.5 (18.0 – 75.5) | 0.050 |
| Macrovascular invasion (-/+) | 171/80 | 101/32 | 70/48 | 0.007 |
| Extrahepatic spread (-/+) | 172/79 | 99/34 | 73/45 | 0.041 |
MV, Muscle volume; M, Male; F, Female; C, Hepatitis C virus, B, Hepatitis B virus; N, NonBnonC; ECOG-PS. Eastern Cooperative Oncology Group performance status; BCLC, Barcelona Clinic Liver Cancer
